# Downregulation of miR-484 is associated with poor prognosis and tumor progression of gastric cancer

**DOI:** 10.1186/s13000-020-00946-8

**Published:** 2020-03-20

**Authors:** Ying Li, Yusong Liu, Jie Yao, Rui Li, Xiaocheng Fan

**Affiliations:** Department of Oncology, Jining Hospital of Traditional Chinese Medicine, Jining, Shandong 272000 China

**Keywords:** Gastric cancer, microRNA-484, Proliferation, Migration, Invasion, Prognosis

## Abstract

**Background:**

Gastric cancer is one of the most common cancers leading to high cancer mortality. MicroRNA-484 (miR-484) has been evaluated as a biomarker for various types of cancers. The subject of this study is to investigate the functional role of miR-484 in gastric cancer.

**Methods:**

The expression of miR-484 in gastric cancer was analyzed by quantitative real-time polymerase chain reaction (qRT-PCR) assay. Kaplan-Meier survival and Cox regression analyses were employed to explore the prognostic significance of miR-484 in gastric cancer. The functional role of miR-484 in gastric cancer was determined by CCK-8 and Transwell assays.

**Results:**

The results showed that miR-484 was significantly downregulated in gastric cancer tissues and cell lines. The downregulation of miR-484 was closely related to differentiation, lymph node metastasis, TNM stage, and poor prognosis. Cox regression analyses demonstrated that miR-484 was an independent prognosis indicator for gastric cancer patients. Additionally, the downregulation of miR-484 enhanced cell proliferation, migration, and invasion in gastric cancer cells.

**Conclusion:**

These data demonstrated that miR-484 can serve as a potential prognostic biomarker and therapeutic target for gastric cancer and it may be involved in the progression of gastric cancer.

## Introduction

Gastric Cancer is a kind of common malignancy with poor prognosis worldwide [1]. A variety of factors are reported to be associated with the development of gastric cancer, such as irregular diet, genetic and epigenetic influence [2]. Due to the lack of typical early symptoms, gastric cancer patients are always diagnosed at advanced stages [3]. The detailed knowledge of the molecular characteristics and the identification of new biomarkers are beneficial for the treatment of gastric cancer. Therefore, finding the biomarker for early diagnosis, prognosis, and treatment of gastric cancer is of great importance.

MicroRNAs (miRNAs, 20–24 nucleotides in length) are a series of non-coding RNAs, which have vital roles in the regulation of gene expression at the post-transcriptional level via binding to the 3′-UTR of target mRNAs [4]. Meanwhile, miRNAs participate in the regulation of a variety of human proteins, which makes it easier to affect the genetic pathways [5]. Currently, miRNA has been reported to act as oncogenes or anti-oncogenes in the initiation and development of various cancers [6–10]. For example, miR-423-3p acts as an oncogene and promotes cell proliferation, migration, and invasion of lung cancer [11]. MiRNAs also have been demonstrated to be implicated in the development, progression, and metastasis of gastric cancer. MiR-196a could promote the invasion and metastasis of gastric cancer by targeting SFRP1; miR-383-5p was downregulated and might act as a tumor suppressor for gastric cancer, and miR-374a has been reported to act as a biomarker for the diagnosis and prognosis of gastric cancer [12–14].

In the present study, miR-484 was investigated in the regulation of gastric cancer. MiR-484 was suggested to play a role in the progression of lung cancer by inhibiting the apoptosis of the cell [15]. It is also reported that miR-484 inhibits cell proliferation of cervical cancer and breast cancer [16, 17]. Additionally, mir-484 has been demonstrated to be down-regulated in gastric cancer [18–20], however, the specific mechanism of miR-484 on the modulation of gastric cancer has not been reported. Therefore, the subject of this study is to investigate the role of miR-484 in the prognosis and progression of gastric cancer, providing considerable therapeutic strategies against gastric cancer.

## Materials and methods

### Patients and tissue samples collection

The paired gastric cancer tissue and matched adjacent normal tissue specimens used for the investigation were collected from 124 patients with gastric cancer admitted to Binzhou Medical University Hospital, from January 2011 to December 2013. The isolated samples were confirmed with pathology diagnosis following the International Union against Cancer. The collected tissues were snap-frozen in liquid nitrogen and stored at − 80 °C for further analysis. Additionally, all the patients had not received any anti-tumor therapies, and a 5-year follow up survey was carried to collect the survival status. Meanwhile, the clinicopathological information of the patients, including age, gender, tumor size, differentiation, lymph node metastasis, and TNM stage are listed in Tables 1 and 2. All participants signed written informed consent in this study. The research was approved by the Ethics Committee of Binzhou Medical University Hospital.

**Table 1:** Correlation between miR-484 expression levels and clinical features in gastric cancer patients

Parameters	Cases No. (*n* = 124)	miR-484 expression		*P*
		Low (*n* = 69)	High (*n* = 55)	
Age				
< 60	63	33	30	0.269
≥ 60	61	36	25	
Gender				
Male	76	37	39	0.632
Female	48	32	16	
Differentiation				
Well + Moderate	71	29	42	0.006
Poor	53	40	13	
T Stage				
T_1_-T_2_	64	34	30	0.388
T_3_-T_4_	60	35	25	
Lauren classification				
Intestinal	68	33	25	0.504
Diffused	56	26	30	
Lymph node metastasis				
Negative	57	24	33	0.015
Positive	67	45	22	
Distant metastasis				
Negative	93	47	46	0.005
Positive	31	22	9	
TNM stage				
I - II	55	26	29	0.002
III - IV	69	43	26

**Table 2:** Multivariate Cox analysis of clinical parameters in relation to overall survival

Characteristics	Multivariate analysis		
	HR	95% CI	*P*
miR-484	2.868	1.148–7.167	0.024
Age	1.176	0.589–2.350	0.645
Gender	1.276	0.611–2.664	0.517
Differentiation	1.563	0.715–3.413	0.263
T stage	1.529	0.760–3.076	0.234
Lauren classification	1.426	0.707–2.879	0.322
Lymph node metastasis	1.844	0.861–3.953	0.116
Distant metastasis	2.172	1.032–4.570	0.041
TNM stage	2.061	1.029–4.126	0.041

### Cell lines and transfection

Gastric cancer cell lines (HGC-27, SNU-1, AGS, NCI-N87) and a normal gastric mucous membrane cell line (GES-1) were purchased from Shanghai Cell Bank of the Chinese Academy of Sciences. All the cells were incubated in the RPMI1640 medium with 10% fetal bovine serum (FBS), in a humidified incubator at 37 °C with 5% CO_2_. For the cell transfection, miR-484 mimic, mimic negative control (NC), miR-484 inhibitor, inhibitor NC (RiboBio, Guangzhou, China) were used for the overexpression and knockdown of miR-484, and the Lipofectamine 2000 Reagent (Invitrogen, USA) was employed according to the instructions of the manufacturer. The sequence of miR-484 mimic is: 5′-UCAGGCUCAGUCCCCUCCCGAU-3′, and the sequence of miR-484 inhibitor is: 5′- AUCGGGAGGGGACUGAGCCUGA-3′.

### RNA extraction and quantitative real-time polymerase chain reaction (qRT-PCR)

The TRIzol reagent (Invitrogen, Carlsbad, CA, USA) was used to extract total RNA of tissues and cells. The extracted RNA reverse transcript to cDNA by a High Capacity cDNA Reverse Transcription Kit (Applied Biosystems, Foster City, CA), and stored at − 20 °C. Next, qRT-PCR was utilized to investigate the expression of miR-484 in tissues and cells, which was carried out with the SYBR Green I Master Mix kit (Invitrogen) and ran on the 7300 Real-Time PCR System (Applied Biosystems, USA). The expression level was determined using the 2^-ΔΔCt^ method and normalized to U6.

### Cell proliferation assay

The transfection cells were seeded into a 96-well plate with a cell concentration of 5 × 10^3^/ mL per well. At 0, 24, 48, 72 h, 10 μL cell counting kit-8 (CCK-8) reagent (Dojindo, Kumamoto, Japan) was added to each well, and then incubated in a humidified incubator for 4 h at 37 °C with 5% CO_2_. The absorbance at 450 nm was measured by a microplate reader (Thermo Fisher Scientific). Experiments were repeated in triplicate.

### Transwell migration and invasion assay

The 24-well transwell chambers (Multiskan MK3, Thermo, Waltham, MA, USA) with a pore size of 8 μm polycarbonate membrane was employed to analyze the migration and invasion. For the migration assay, the transfected cells with the concentration of 2 × 10^5^/ml were seeded into the upper compartment and then incubated in the culture medium without FBS at 37 °C for 24 h. While for the invasion assay, the upper chamber was coated with Matrigel (BD Biosciences, Franklin Lakes, NJ, USA). The lower chamber was filled the culture medium with 10% FBS as a chemoattractant. After incubating for 48 h, the migrated cells were stained with 0.1% crystal violet and counted by a microscope.

### Statistical analysis

All results are presented as mean ± standard deviation (SD). Statistical analysis of the data was conducted with SPSS 20.0 software (SPSS, Inc., Chicago, IL, USA) and GraphPad Prism 5.0 software (GraphPad Software, Inc., Chicago, USA). The differences between the two groups are analyzed through Student’s t-test. The correlation of miR-484 with clinicopathological features was evaluated by χ^2^ test. Kaplan-Meier analysis and Cox regression analysis were used to determine the prognostic significance of miR-484. The differences were considered to be significant when *P* < 0.05.

## Results

### Expression of miR-484 in gastric cancer tissues and cell lines

**Fig. 1:**
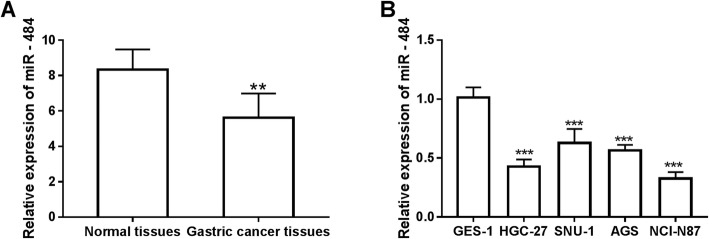
Expression of miR-484 in gastric cancer tissues and cell lines. **a**. Expression of miR-484 was downregulated in gastric cancer tissues compared with adjacent normal tissues. (**P < 0.01, ***P < 0.001). **b**. Expression of miR-484 was decreased in gastric cancer cell lines (HGC-27, SNU-1, AGS, NCI-N87) compared with normal cell line GES-1. (***P < 0.001)

The expression of miR-484 was analyzed by qRT-PCR in gastric cancer tissues and adjacent normal tissues. As shown in Fig. 1a, compared with adjacent normal tissues, miR-484 in gastric cancer tissues was significantly downregulated (*P* < 0.001). Additionally, the expression of miR-484 in four different gastric cancer cell lines and a normal cell line was investigated. The result showed that the expression of miR-484 in gastric cancer cell lines was lower than that in the normal cell lines, and the difference was significant (*P* < 0.001, Fig. 1b). The expression of miR-484 was different in four cancer cell lines, with the lowest expression appeared in NCI-N87 cell line, followed by HGC-27 cell line, and the expression of miR-484 in AGS and SNU-1 was a bit higher than that in the other two cell lines. Due to the relatively low expression, NCI-N87 and HGC-27 were selected for the following experiments.

### The relationship between the expression of miR-484 and the clinicopathological features of gastric cancer patients

According to the mean expression level of miR-484 (5.617), all 124 gastric cancer patients were divided into two groups: high expression group and low expression group. Next, the relationship between the expression of miR-484 and the clinicopathological features of gastric cancer patients was evaluated through χ^2^ test, the results are summarized in Table 1. The results revealed that the expression of miR-484 was significantly related to differentiation (*P* = 0.006), lymph node metastasis (*P* = 0.015), distant metastasis (*P* = 0.005) and TNM stage (*P* = 0.002). Nevertheless, the age and gender of patients were not associated with the expression of miR-484 (all *P* > 0.05).

### Prognostic value of miR-484 in gastric cancer

**Fig. 2:**
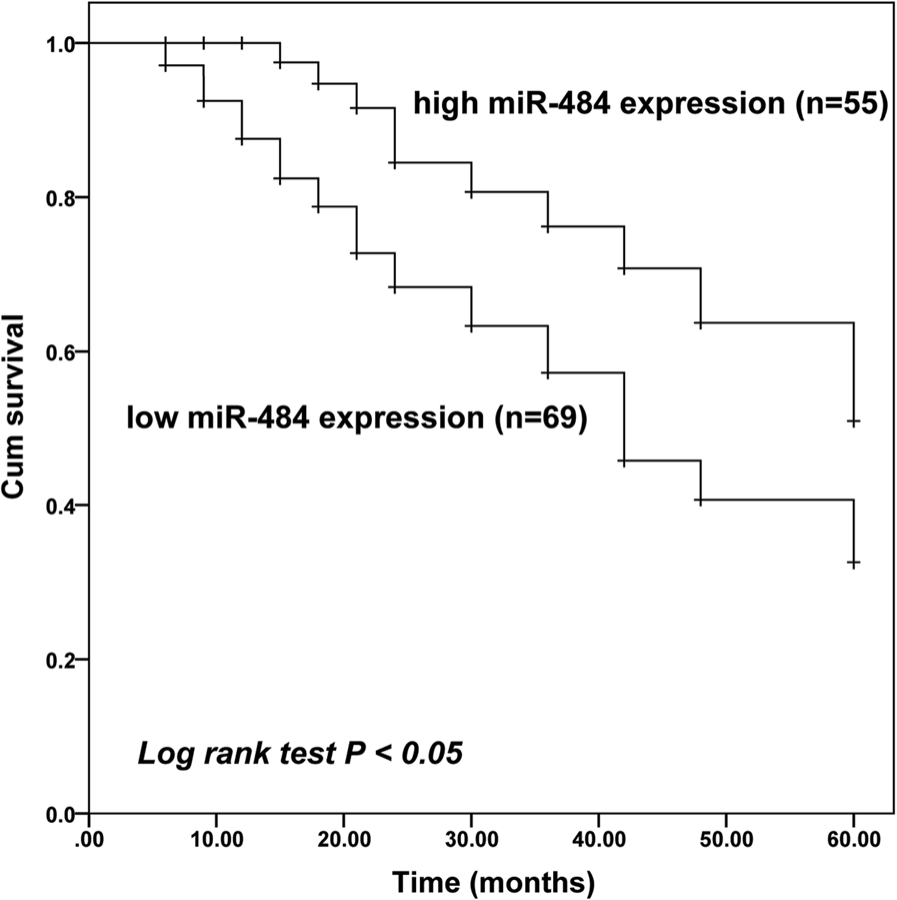
Kaplan-Meier survival curves for gastric cancer patients based on the expression of miR-484. *P* value was calculated using the log-rank test. Patients with low miR-484 expression had a shorter survival time than those with high miR-484 expression

Based on the expression of miR-484 and the overall survival information of gastric cancer patients from a 5-year follow up survey, the Kaplan-Meier analysis was employed to assess the role of miR-484 in the prognosis of gastric cancer. As shown in Fig. 2, the overall survival rate of patients with high expression of miR-484 was much higher than those with low expression of miR-484 (log-rank test *P* = 0.001). Furthermore, the multivariate Cox’s hazard regression analysis was used to investigate the effect of miR-484 and other clinic clinicopathological features on the survival rate of gastric cancer patients. Results showed that the expression of miR-484 (HR = 2.868, 95% CI = 1.148–7.167, *P* = 0.024), distant metastasis (HR = 2.172, 95% CI = 1.032–4.570, *P* = 0.041) and the TNM stage (HR = 2.061, 95% CI = 1.029–4.126, *P* = 0.041) of patients are independent prognostic factors for 5-year overall survival in gastric cancer patients (Table 2).

### The role of miR-484 in cell proliferation, migration, and invasion of gastric cancer

**Fig. 3:**
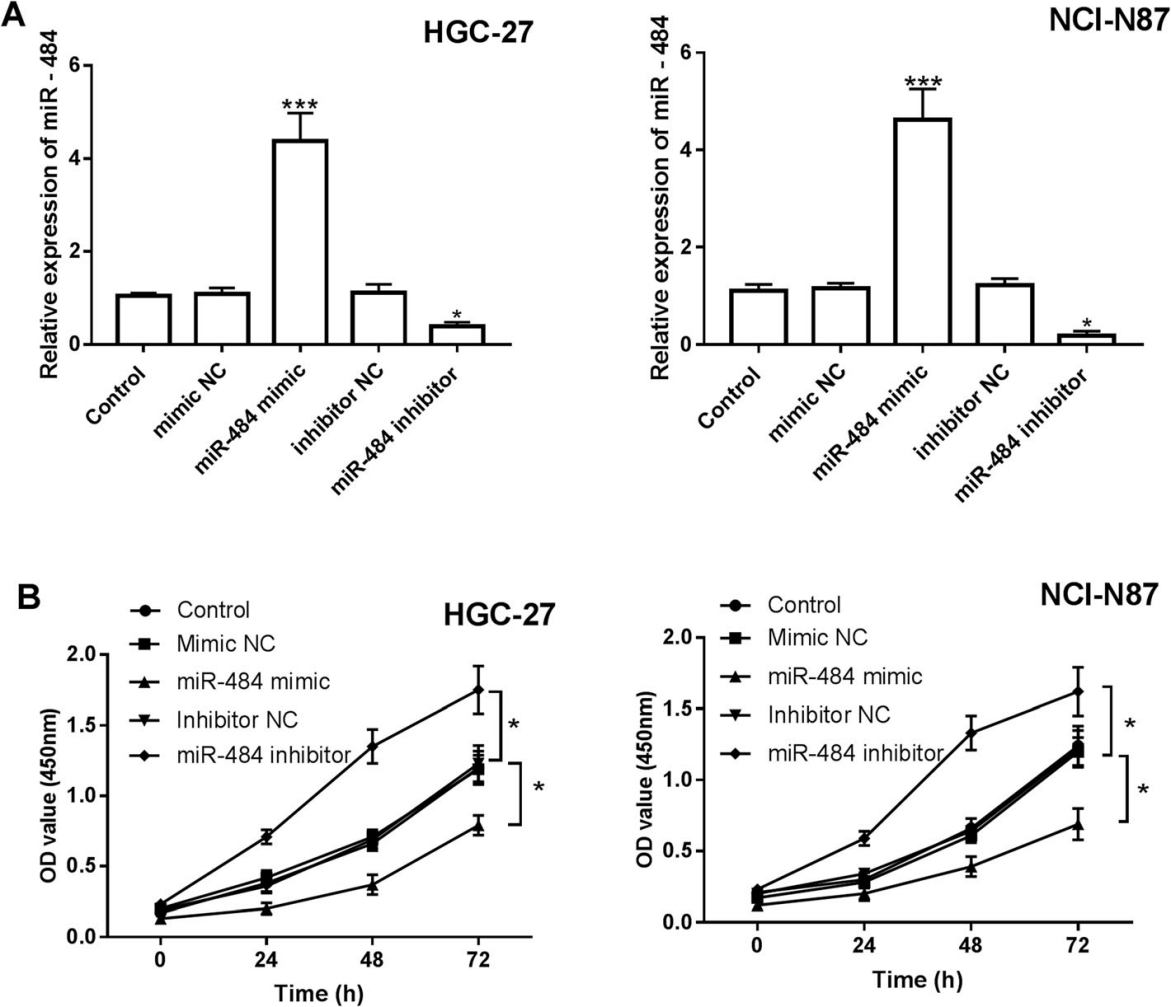
Effect of miR-484 on the cell proliferation of HGC-27 and NCI-N87. **a**. Expression of miR-484 in the presence of miR-484 mimic or miR-484 inhibitor (*P < 0.05, ***P < 0.001). **b**. Overexpression of miR-484 inhibited the proliferation of gastric cancer cells (*P < 0.05)

**Fig. 4:**
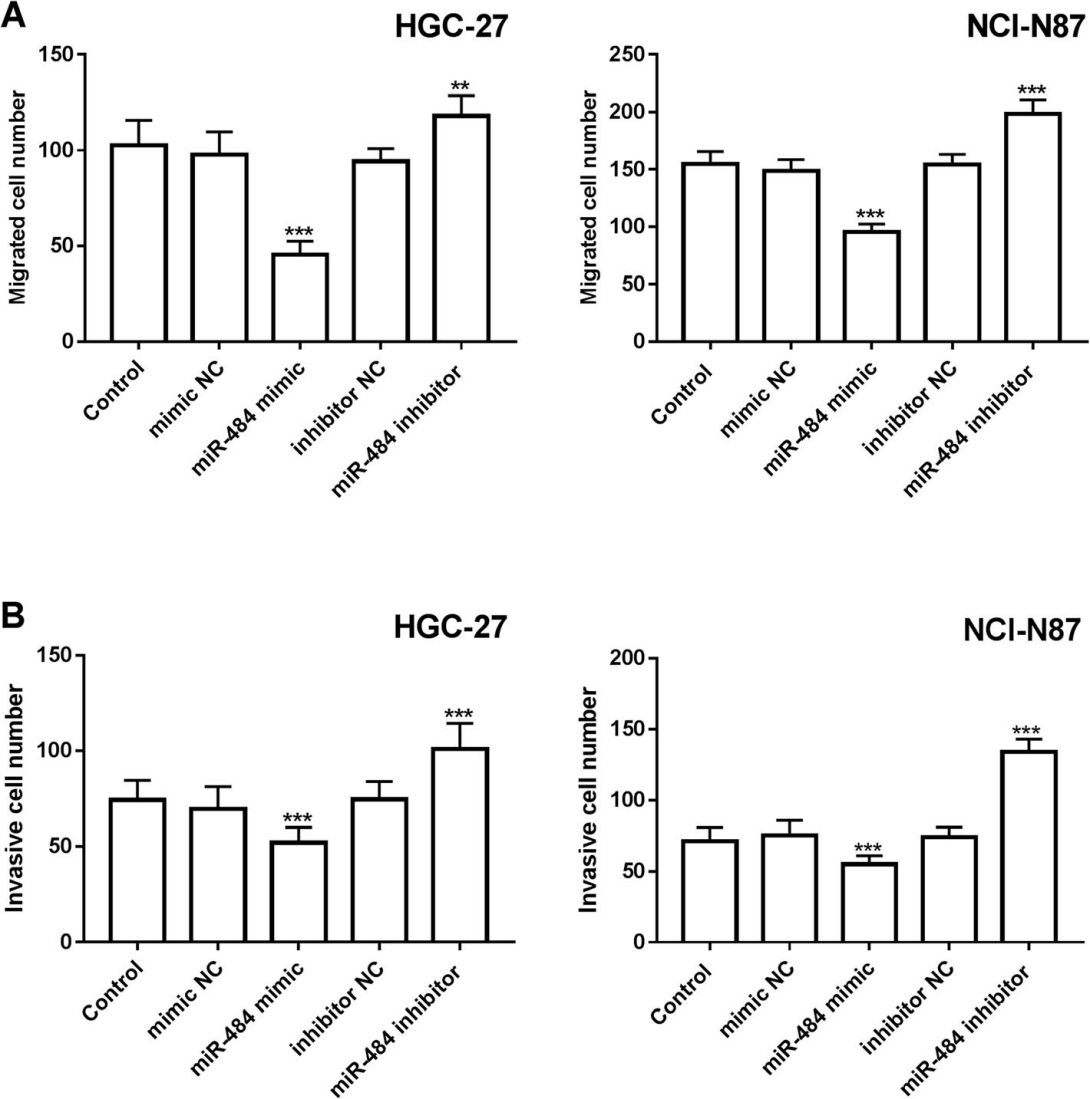
Effect of miR-484 on the migration and invasion of HGC-27 and NCI-N87. **a**. Knockdown of miR-484 enhances the migration of gastric cancer cells (**P < 0.01, ***P < 0.001). **b**. Reduction of miR-484 promotes the invasion of gastric cancer cells (***P < 0.001)

The HGC-27 and NCI-N87 were transfected with miR-484 mimic or miR-484 inhibitor to evaluate the functional role of miR-484 in gastric cancer. The results in Fig. 3a showed that miR-484 mimic increased the expression of miR-484, and miR-484 was downregulated in the presence of miR-484 inhibitor, the difference was significant (*P* < 0.01). Then the cell proliferation capacity was evaluated through the CCK-8 assay. The proliferation of HGC-27 and NCI-N87 with high miR-484 expression was inhibited, while the cell lines with low miR-484 expression were promoted, and the difference was significant (*P* < 0.05, Fig. 3b). Meanwhile, the migration and invasion of the cell lines were also investigated. The results suggested that the high expression of miR-484 increased by miR-484 mimic led to an inhibition of migration, whereas the downregulation of miR-484 inhibited by miR-484 inhibitor enhanced the migration, and the same effects were exerted on the invasion of the cell lines (*P* < 0.01, Fig. 4a and b).

## Discussion

Gastric cancer is considered to be a highly lethal malignancy, due to its terrible proliferation, migration, and invasion, which can lead to high mortality and poor prognosis. The modular and cellular factors that governing these processes remain unclear [21]. Multiple studies have reported that microRNAs could regulate target mRNAs and its abnormal expression was closely related to the migration, invasion, and proliferation of various cancers [10, 22–24]. For instance, miR-593 can inhibit the migration and invasion of non-small cell lung cancer cells by targeting SLUG-associated signaling pathways [25]. MiRNAs have different behaviors in various cancers. MiR-601 acts as an oncogene in gastric cancer, which was upregulated and related with the poor prognosis of gastric cancer [26]. On the other hand, miR-601 was also a prognosis marker of breast cancer, because its downregulation could promote the proliferation, migration, and invasion of breast cancer cells [27]. For gastric cancer, a variety of miRNAs have been reported to play a role in its proliferation, migration, and invasion, such as miR-4317, miR-501-5p, miR-214 and so on [28–30]. Moreover, miR-214 was also demonstrated to enhance the invasion of breast cancer cells, and it was upregulated in breast cancer, which is opposite to the expression in gastric cancer [31].

In the present study, the role of miR-484 in the proliferation, migration, and invasion of gastric cancer was studied, and we found the expression of miR-484 was downregulated in gastric cancer tissues compared with paired adjacent normal tissues. Moreover, the expression of miR-484 was associated with differentiation, lymph node metastasis, and TNM stage. From these results, it can be inferred that miR-484 may be a tumor suppressor, which is consistent with previous studies [18–20]. Furthermore, the association of miR-484 with tumor differentiation, lymph node metastasis and TNM stage of the patients was found, indicating the potential role of miR-484 in the development of gastric cancer. Additionally, based on the survival information and the clinical features of patients, the Kaplan-Meier curve and Cox regression analyses were employed to explore the clinical significance of miR-484. From the results, we found that the patients with low expression of miR-484 had shorter survival time, which indicated that the downregulation of miR-484 was associated with poor overall survival.

Previous studies have demonstrated the dysregulation of miRNAs that play roles in cell proliferation, migration, and invasion of various cancers. For example, the downregulation of miR-449a exerted inhibitory effect on the proliferation, migration, and invasion of cervical cancer [8]. The overexpression of miR-200 could suppress tumor proliferation in colorectal cancer [32]. Actually, the role of miR-484 in a certain type of cancer is still a controversial issue. Recent research has found the dysregulation of miR-484 in various cancers, and its expression was different. The expression of miR-484 in lung cancer was increased, but it was downregulated in cervical cancer and metastatic renal cell carcinoma [16, 33, 34]. It also has been reported that miR-484 was highly specific for prostate cancer, which could make it act as a prostate cancer screening biomarker [35]. In hepatocellular carcinoma, miR-484 has an oncogenic role by targeting TUSC5 [36]. Moreover, miR-484 could act as the biomarker for many other cancers, including lung cancer, breast cancer, cervical cancer and colorectal cancer [15, 16, 37]. In the present study, the upregulation of miR-484 inhibited the proliferation, migration, and invasion of gastric cancer cells, which suggested that miR-484 might be involved in the progression of gastric cancer.

However, there are also some limitations in our study. Previous studies have demonstrated that miR-484 can reduce the expression of a series of oncogenes, such as ZEB1, SMAD2, etc., inhibit the epithelial-mesenchymal transition (EMT) of tumor cells, or regulate the extracellular signal-regulated kinase 1/2 signaling [16, 34, 38]. The precise molecular mechanisms underlying the role of miR-484 should be investigated in further studies. On the other hand, only a limited number of samples were included in the current study and increased sample size should be used in the future to validate the accuracy of miR-484 as a biomarker.

To summarize, the present study indicated the downregulation of miR-484 was associated with the poor overall survival of gastric cancer patients, which makes it possible to act as a candidate prognostic biomarker. The overexpression of miR-484 in gastric cancer leads to inhibited cell proliferation, migration, and invasion, indicating that miR-484 may be a therapeutic target for the treatment of gastric cancer.

## Data Availability

The datasets used during the current study are available from the corresponding author on reasonable request.

## Abbreviations

miR-484: microrna-484
qRT-PCR: Quantitative real-time polymerase chain reaction
miRNAs: MicroRNAs
FBS: Fetal bovine serum
NC: Negative control
CCK-8: Cell counting kit-8
SD: Standard deviation
EMT: Epithelial-mesenchymal transition
